# A rare cause of right iliac fossa pain

**DOI:** 10.4103/0971-9261.54812

**Published:** 2009

**Authors:** Mansoor Khizer, Samujh Ram, Abdul Mannan Khan

**Affiliations:** Department of Pediatric Surgery, Riyadh Medical Complex, Riyadh, Kingdom of Saudi Arabia; 1 Department of Pediatric Radiology, Riyadh Medical Complex, Riyadh, Kingdom of Saudi Arabia; 2 Department of Pediatric Surgery, Advanced Pediatric Center, Post Graduate Institute of Medical Education and Research, Chandigarh 160 012, India

**Keywords:** Acute appendicitis, appendectomy, torsion appendix epiploicae

## Abstract

A child with torsion of appendix epiploicae presenting as acute right iliac fossa pain in abdomen is reported.

## INTRODUCTION

Right iliac fossa pain is commonly diagnosed as acute appendicitis. We report a case presenting as acute appendicitis who actually had torision of an appendix epiploicae and gangrene. A brief review of literature for this relatively rare condition is presented.

## CASE REPORT

A 12-year-old male obese child presented with a 1 day history of severe abdominal pain and fever. The pain was sudden in onset and severe in intensity. It started from the right iliac fossa and remained localized without any radiation. No relationship to intake of food or any posture was stated. It was continuous and pricking in nature. The patient also had a low-grade fever, which was continuous. This pain was associated with nausea but no vomiting. The patient had significant anorexia. There were no urinary or bowel complaints. There was no significant history of similar pain and the patient had enjoyed good general health before.

Upon examination, he was an obese child in obvious distress lying in bed. His pulse was 130/min, his blood pressure was 110/65, and his temperature was 38°C. The rest of the general physical examination was normal. The abdominal examination showed tenderness in the right iliac fossa with rebound tenderness but no guarding or rigidity. There was no organomegaly and bowel sounds were present with an unremarkable rectal digital examination. The patient weighed 80kg.

Investigations revealed a white blood cell (WBC) count of 11.5 K/uL and hemoglobin was 14.4 g/dl. The rest of the serum chemistry values were normal. A plain abdominal radiograph was unremarkable and an ultrasound showed only minimal free fluid.

**Figure 1:**
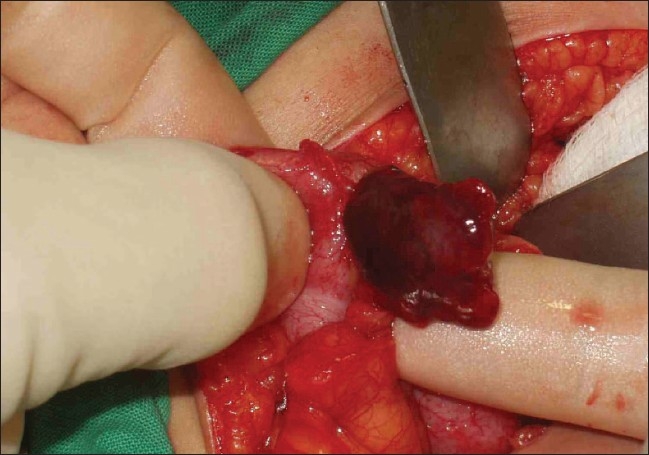
Torsed gangrenous appendix epiploicae

**Figure 2:**
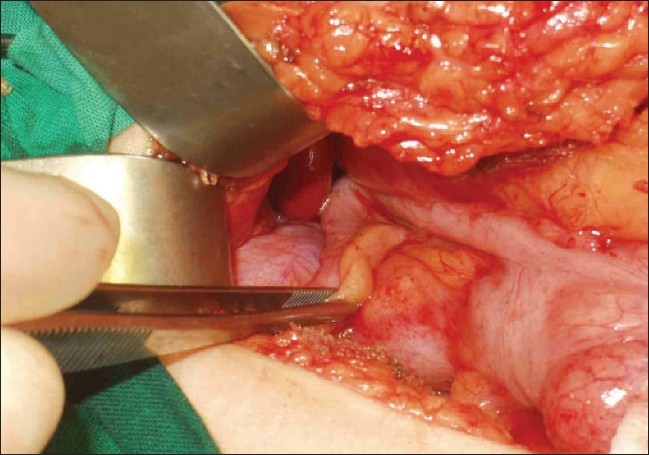
Minimally inflammed appendix

With a provisional diagnosis of acute appendicitis, the patient was taken to the operating room for an appendectomy. Upon opening of the peritoneal cavity, lightly hemorrhagic fluid was seen. On handling of the cecum, a torsed, gangrenous appendix epiploicae with 2 complete twists of the base was found [Figure 1]. It was transfixed and excised. The appendix was retrocecal in position and subhepatic in location with minimal inflammation and was kinked [Figure 2]. An appendectomy was also done.

The patient had an uneventful recovery and was discharged 2 days after the surgery. A peritoneal swab was negative for any growth. The histopathology was consistent with gangrenous appendix epiploicae.

## DISCUSSION

Torsion of the appendix epiploicae is a rare condition that may present as acute right iliac fossa pain and usually mimics an acute appendicitis.[1] It was first reported by Marchett in 1851.[2] In a large series, the male to female ratio was 2:1.[3] Obesity has been consistently found to be a predisposing factor.[4] The patient can present with irritating voiding symptoms or hematuria.[5] It is relatively rare in younger children because of the relative paucity of omental fat.[6] It may occur because of an abnormal congenital attachment of the appendices.[7] Diseases of the greater omentum include mostly torsion followed by idiopathic infarction and most rarely primary omentitis.[8] In one series, all patients had torsion on the right side with 360-720° torsion and hemorrhagic peritoneal effusion was a consistent feature.[9] An abdominal ultrasound usually shows localized omental thickening.[1] A contrast-enhanced computed tomography (CT) scan shows a whirling pattern of fatty streaks within the greater omentum.[10] Diagnosis is rarely made pre-operatively and if the appendix is found to be normal, torsion of the appendix epiploicae should be considered.[11] According to one series, the pathology should be dealt with by emergency surgery by removal of the torsed appendix, closure of the bed with a double row of interrupted sutures, and coverage by a pedicled omental graft.[12] Most commonly a laparoscopy is both diagnostic and therapeutic and open surgery can be avoided.[1] If torsion is confidently diagnosed, it responds well to symptomatic conservative treatment as well.[13]

Our patient was classically obese and presented with a very typical history. No significant ultrasound findings were present except for free fluid. However, the presence of hemorrhagic effusion at the time of the laparotomy was highly suspicious of the diagnoses of torsion.

In overweight children with an acute onset history of abdominal pain mimicking acute appendicitis, torsion of appendix epiploicae should be considered in a differential diagnosis. Attempts should be made to confirm the diagnosis preoperatively so that a trial of conservative treatment can be given. In a case requiring intervention, a laparoscopy offers both diagnostic and therapeutic advantage over open surgery.
